# Speed invariant gait recognition—The enhanced mutual subspace method

**DOI:** 10.1371/journal.pone.0255927

**Published:** 2021-08-11

**Authors:** Yumi Iwashita, Hitoshi Sakano, Ryo Kurazume, Adrian Stoica

**Affiliations:** 1 Jet Propulsion Laboratory, California Institute of Technology, Pasadena, CA, United States of America; 2 Kyushu University, Fukuoka, Japan; 3 Shimane University, Matsue, Shimane; IRCCS E. Medea, ITALY

## Abstract

This paper introduces an *enhanced MSM* (Mutual Subspace Method) methodology for gait recognition, to provide robustness to variations in walking speed. The *enhanced MSM (eMSM)* methodology expands and adapts the MSM, commonly used for face recognition, which is a static/physiological biometric, to gait recognition, which is a dynamic/behavioral biometrics. To address the loss of accuracy during calculation of the covariance matrix in the PCA step of MSM, we use a 2D PCA-based mutual subspace. Furhtermore, to enhance the discrimination capability, we rotate images over a number of angles, which enables us to extract richer gait features to then be fused by a boosting method. The eMSM methodology is evaluated on existing data sets which provide variable walking speed, i.e. CASIA-C and OU-ISIR gait databases, and it is shown to outperform state-of-the art methods. While the enhancement to MSM discussed in this paper uses combinations of 2D-PCA, rotation, boosting, other combinations of operations may also be advantageous.

## Introduction

Biometrics authentication has provided a large number of opportunities for security systems and forensic applications. Gait belongs to the class of “behavioral” biometrics. Gait-based person recognition does not require interaction with the subjects, and works from a distance, thus making it attractive to law enforcement agencies. For example, in the United Kingdom a gait recognition system used CCTV imagery to provide court evidence in a case of burglary [1].

Gait recognition methods which extract features from images captured by cameras have produced good results. Several techniques to extract gait features were proposed, such as gait energy image (GEI) [2], affine moment invariants [3], active energy image [4], gait flow image [5], and frame difference frieze pattern [6]. The first two techniques can be considered as gait features containing human shape information rather than motion information, while the last two techniques extract motion information explicitly. Among them, GEI, which is the average image of silhouette images for a duration of one gait cycle, has received the most attention due to its high performance and simple implementation. However, since image-based gait recognition is sensitive to changes in appearance due to various reasons, such as clothing and walking direction changes, the correct classification rate is low in cases where the subject’s appearance is different from that in the database. Such low performance may occur even when the subject’s walking speed is different from that in the database.

The walking speed change causes variations in pitch and stride, which result in non-invariant gait features. Various approaches have been proposed to tackle this issue [7, 8], however the performance of these methods is not sufficient. For example, with a challenging data set such as OU-ISIR Gait Speed Transition Dataset [8], Mansur et al. reported a correct classification ratio of 84 [%]. This leaves 16 [%] misclassification, which is still high. Walking speed can change during motion and it may be consciously modified to adapt to the context—such as slowing down when approaching a red light or accelerating when there are only a few seconds to finish crossing an intersection. It would thus be useful to extract motion-invariant information.

In a preliminary study we proposed to use a mutual subspace method (MSM) [9, 10], which is an image set-based matching approach used for face recognition (a static/physiological biometric) to capture speed-invariant information [11]. This method involves dividing the human body area in multiple regions and use local movement information [12]. The preliminary experimental evaluations on a challenging gait data set for speed transition (OU-ISIR Gait Speed Transition Dataset [8], OU-ISIR Treadmill Dataset A [13], and CASIA-C Dataset [14]) had shown promise [11, 12], however the performance of the MSM-based method diminishes when the speed mismatches are large.

Time-series images of each class in a gallery dataset are given as input into MSM, and a model of each class is obtained as a linear subspace. The same procedure is applied to images in an input probe dataset. To obtain the model, 2D images are first transformed into vectors, and principal component analysis (PCA) is applied to calculate subspaces from the vectors. Here, we refer to both PCA and the spectrum decomposition (autocorrelation-based PCA) as PCA. Similarities between gallery and input subspaces are calculated as squared cosines of canonical angles between two subspaces, and these similarities are used for recognition. However, as Yang et al. pointed out [15], due to the large sizes of the vectors, the covariance matrix calculated in the process of PCA can not be calculated accurately. This is the cause for performance decrease in gait recognition.

In this paper we propose an enhanced MSM to improve accuracy and robustness to speed by the following contributing enhancements:

- The use of 2D PCA [15] to calculate the covariance matrix with high accuracy, which enables us to represent subspaces of the original images more efficiently than 1D PCA (denoted as 2D-PCA MSM).
- The use of rotated images, and applying 2D-PCA MSM to rotated images, to extract rich gait information (denoted as 2D-PCA-R MSM (2D-PCA-Rotated MSM)).
- The use of boosting, to adaptively fuse similarities of canonical angles from 2D-PCA-R MSM (denoted as the B-2D-PCA-R MSM (Boosted-2D-PCA-R MSM)).
- We demonstrate the improved performance and robustness of the eMSM (enhanced MSM) compared with state-of-the-art methods with three challenging datasets (OU-ISIR Treadmill Dataset A, OU-ISIR Gait Speed Transition Dataset, and CASIA-C Dataset).

## Related work

### Gait recognition robust to speed variations

Existing gait recognition approaches robust to speed variations can be categorized into 2 approaches: (i) transforming the gait features from various speeds into a common walking speed and (ii) extracting speed-invariant gait features. In the “common speed” approach, Tanawongsuwan and Bobick proposed a stride normalization procedure for double-support frames [7], and Kusakunniran et al. proposed a gait shape description, which conserves discriminative information in the gait signature [16].

In the “speed-invariant features” approach, various methods have been proposed to extract speed-invariant gait features [11, 12, 17–19]. Xu et al. proposed a method based on single-support gait images, whose appearance is not changed by speed variations [18]. Guan and Li employed Random Subspace Method (RSM)-based method [17], which reduces the generalization errors by combining a large number of weak classifiers. Khan et al. proposed a spatiotemporal motion-based method [19], which is not affected by appearance changes.

In a preliminary study, we proposed a mutual subspace method (MSM)-based approach to be robust to speed variations [11], with work continued in [12] by dividing the human body area in multiple regions, followed by adaptive choice of areas that have high discrimination capability. In the case that the MSM-based method with divided areas [12] is applied to relatively high resolution gait images (e.g. the human height is more than 100 pixels), we achieved better performance than the MSM-based method [11]. However, in the case of low resolution images, the performance of the MSM-based method with divided areas degrades, due the loss of discrimination capability in each divided area.

One of the common issues in existing approaches is that the performance suffers from large speed mismatch. The enhanced MSM proposed in this paper addresses this challenge and achieves state-of-the-art performance in speed invariant gait recognition.

### Deep learning-based gait recognition

Deep learning-based approaches have been applied to gait recognition and achieved state-of-the-art performance [20, 21]. Most of the methods focus on view-invariant gait recognition, since many data sets contain large view variance [22]. However, these approaches are not suitable for use with small data sets.

## Mutual subspace method (MSM)

This section reviews MSM’s application to gait recognition and MSM. It explains a relation between MSM and GEI, followed by the rationale for why MSM works well in gait recognition.

### The use of MSM in gait recognition

**Fig 1:**
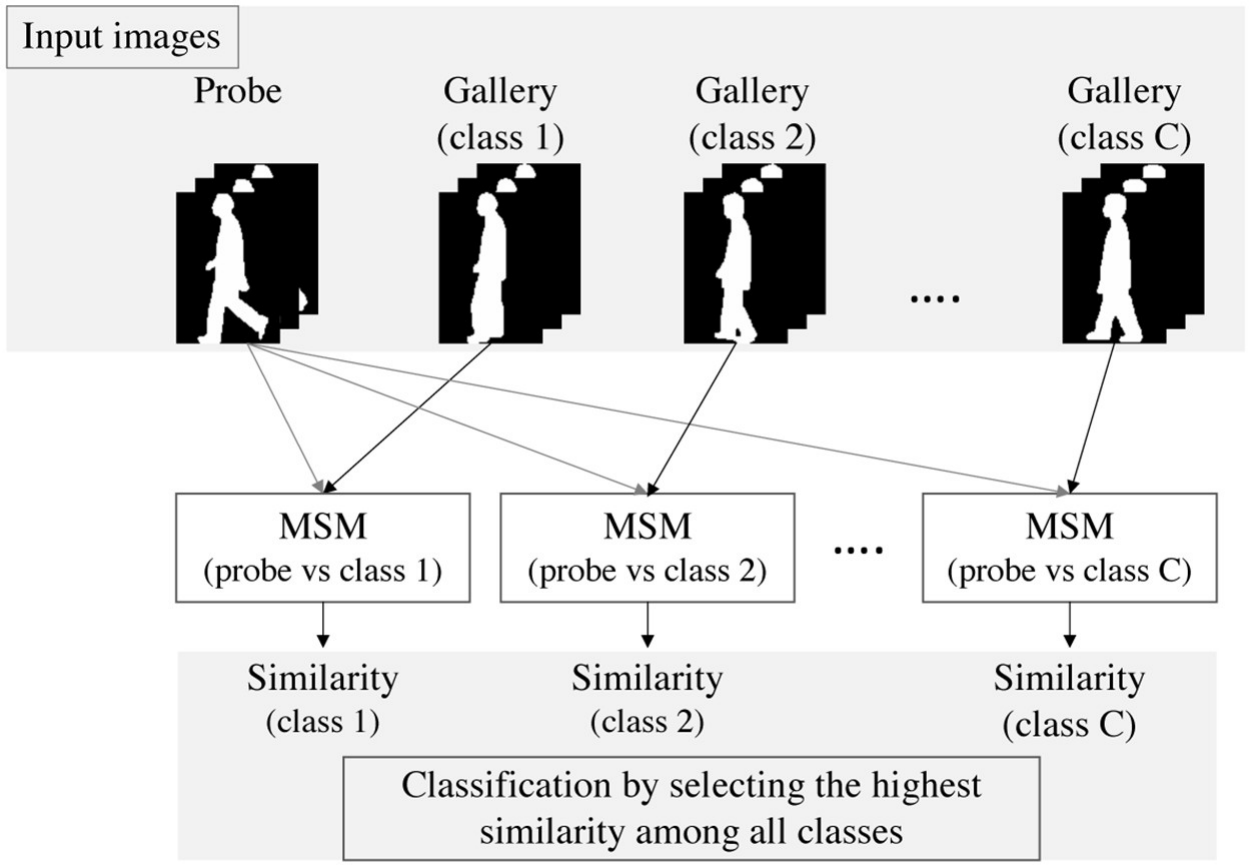
An overview of the gait recognition based on MSM. From gallery images of class *c* and input images of a probe subject, similarities between the class *c* and the subject are calculated based on MSM. This process is repeated for all classes in the database, and the subject is classified based on the similarities.

An overview of the gait recognition based on MSM is shown in Fig 1. Assume that there is a person (probe) to be recognized and that there are multiple people registered in the database as a gallery data set. To recognize the person with MSM, time-series gait silhouette images are used as input. In general each silhouette area is scaled to a uniform height. From gallery images of class *c* and input images of the person, similarities between the class *c* and the subject are calculated based on MSM. This process is repeated for all classes in the database.

In this paper we use the following definition to calculate correct classification and misclassification rates. The person is classified by choosing highest similarities among all classes. This classification process is applied to all people in the probe dataset, and the numbers of true positives (*TP*) and of false positives (*FP*) are obtained. The correct classification and misclassification rates are calculated by *TP*/(*TP* + *FP*) and *FP*/(*TP* + *FP*), respectively.

### Mutual subspace method (MSM)

MSM is an image set-based matching approach. MSM models template images of each class in a gallery dataset and input images in a probe dataset as subspaces. The similarity measure in MSM is calculated as a cosine squared canonical angle between each of the gallery subspaces and the input subspace. Details of the MSM are as follows.

Let us assume a *C* class pattern recognition problem. 2D images $I_{c,n}^{G}$ ($1\leq n\leq N_{c}^{G}$, where $N_{c}^{G}$ is the number of images in class *c* in the gallery (G) dataset) are transformed into 1D vectors $x_{c,n}^{G}$. Eigenvalues $\lambda_{c}^{d^{G}}$ and eigenvectors $\phi_{c}^{d^{G}}$ of the class *c* gallery samples (1 ≤ *d*^*G*^ ≤ *D*^*G*^, where *D*^*G*^ is the dimensionality of the gallery subspace) are obtained from a class autocorrelation matrix $\Gamma_{c}^{G}$ as

$$\begin{array}{c} \Gamma_{c}^{G}=\frac{1}{N_{c}^{G}}\sum_{n=1}^{N_{c}^{G}}\sum_{l=1}^{N_{c}^{G}}x_{c,n}^{G}{\left(x_{c,l}^{G}\right)}^{T}. \end{array}$$ (1)

The same procedure is applied to input images $I_{n}^{P}$ (1 ≤ *n* ≤ *N*^*P*^, where *N*^*P*^ is the number of images of each subject in the probe (P) dataset) to obtain eigenvectors $\psi^{d^{P}}$ (1 ≤ *d*^*P*^ ≤ *D*^*P*^, where *D*^*P*^ is the dimensionality of the input subspace). The similarities in MSM, which are squared cosines of canonical angles between two subspaces, are calculated as eigenvalues of the following matrix [23].

$$\begin{array}{c} Z_{c}=\left(\zeta_{c}^{i,j}\right),\;\;\zeta_{c}^{i,j}=\sum_{d=1}^{d^{P}}\left(\phi_{c}^{i}\cdot \psi^{d}\right)\left(\psi^{d}\cdot \phi_{c}^{j}\right). \end{array}$$ (2)

We define the similarities as $s_{c}^{\left(r,d^{G},d^{P}\right)}$ (1 ≤ *r* ≤ *R* where *R* is the maximum number of canonical angles to be used in the recognition process and less than or equal to the rank of ***Z***_*c*_. In existing methods *r* = 1 is commonly used). Intuitively, canonical angles between the gallery and input subspaces are regarded as the angles which minimize appearance difference between the gallery and input data. Eigenvectors $\omega_{c}^{\left(r,d^{G},d^{P}\right)}$ are also calculated.

Dimensionalities of gallery and input subspaces, *d*^*G*^ and *d*^*P*^, are influential parameters. Roughly speaking the dimensionality is proportional to variations in gallery and input images. However when the dimensionality increases too much, the recognition accuracies got worse, because of the increasing amount of intersections among subspaces. Generally such dimensionalities are defined by cross validation experiments.

### A relation between MSM and GEI

**Fig 2:**
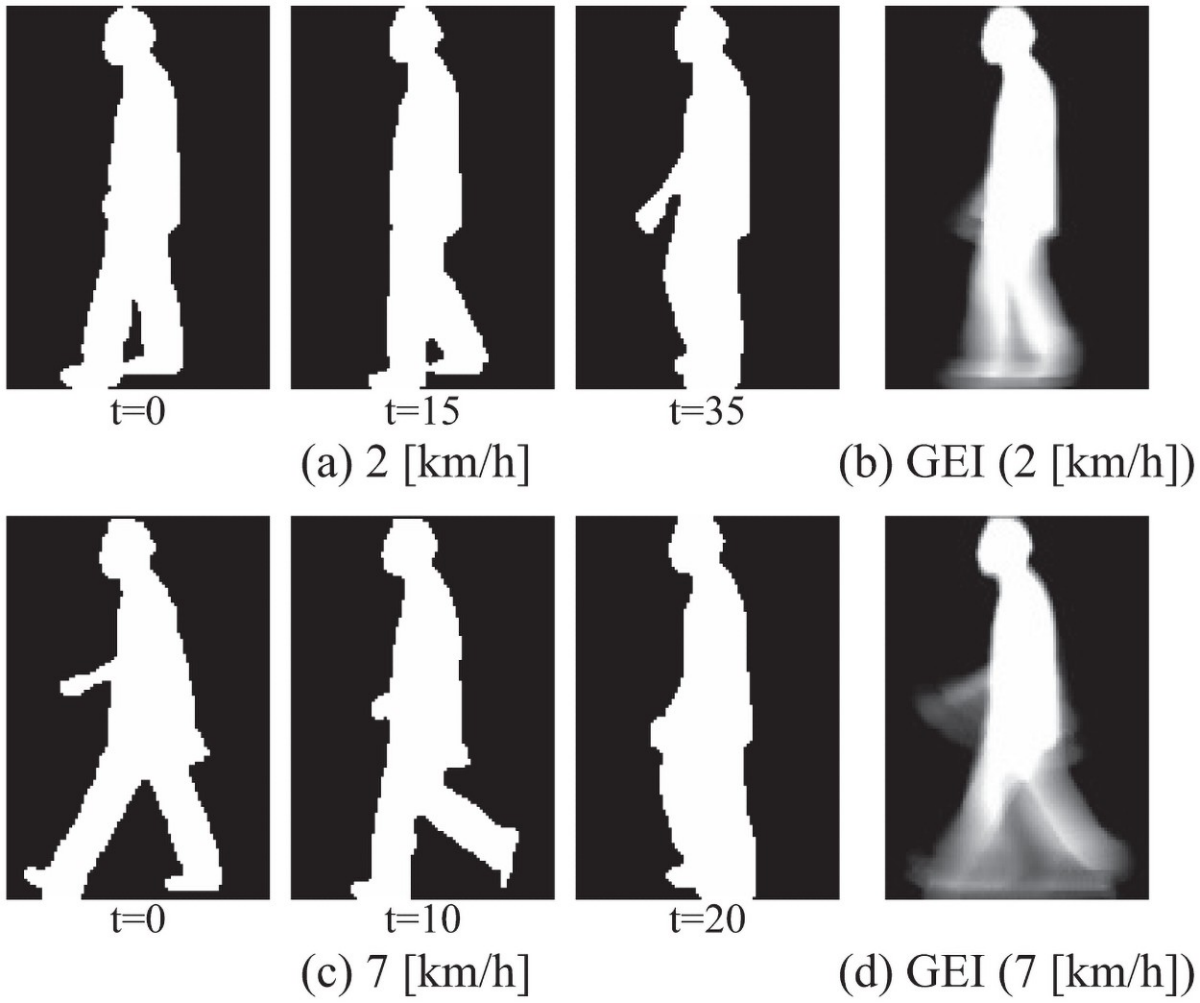
(a) Examples of gait images (2 [km/h]) in OU-ISIR Treadmill Dataset A [13], (b) GEI of (a), (c) examples of gait images (2 [km/h]), and (d) GEI of (c).

**Fig 3:**
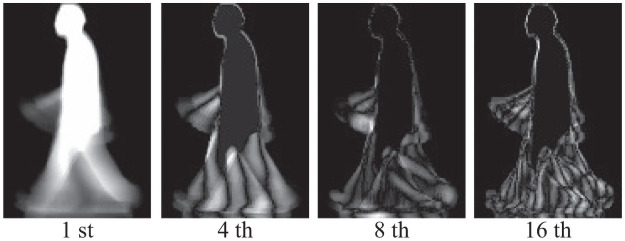
Visualizations of eigenvectors *ϕ*^*c*^ (1st, 4th, 8th, and 16th) of MSM. The image of the first principal component is similar to GEI.

Fig 2(a) and 2(c) show example images of two different walking speeds (2 [km/h] and 7 [km/h]) from the OU-ISIR Treadmill Dataset A, explained in details in section of experiments. Fig 3 shows visualizations of eigenvectors ***ϕ***_*c*_ (e.g. 1st, 4th, 8th, and 16th) of 7 [km/h] (Fig 2(c)). These images suggest that the first principal component represents human shape, such as head and torso, and the following principal components show motion information.

Fig 2(b) and 2(d) show the GEI of 2 [km/h] and 7 [km/h], respectively. From a series of silhouette images ***I***_*n*_ (1 ≤ *n* ≤ *N*, where *N* is the number of images), GEI is calculated as $\frac{1}{N}\sum_{n=1}^{N}I_{n}$. Compared with Fig 2(d) and the visualization of the 1st eigenvector in Fig 3, we can see both are very similar. From a linear algebra perspective, GEI can be regarded as a representation of gait images with a 0-dimensional affine subspace. In the definition of an affine space, a point is defined as zero-dimensional. GEI is in a 0-dimensional subspace, since at each pixel a value is calculated as an average of time-series values (i.e. removing time information). The GEI-based gait recognition method can be interpreted as a subset of class-featuring information compression (CLAFIC) [24], which is one of the subspace methods with 0-dimensional affine subspace. Thus it is natural to assume that a d-dimensional subspace may perform better than the 0-dimensional one (i.e. GEI). MSM is categorized as the d-dimensional subspace method.

**Fig 4:**
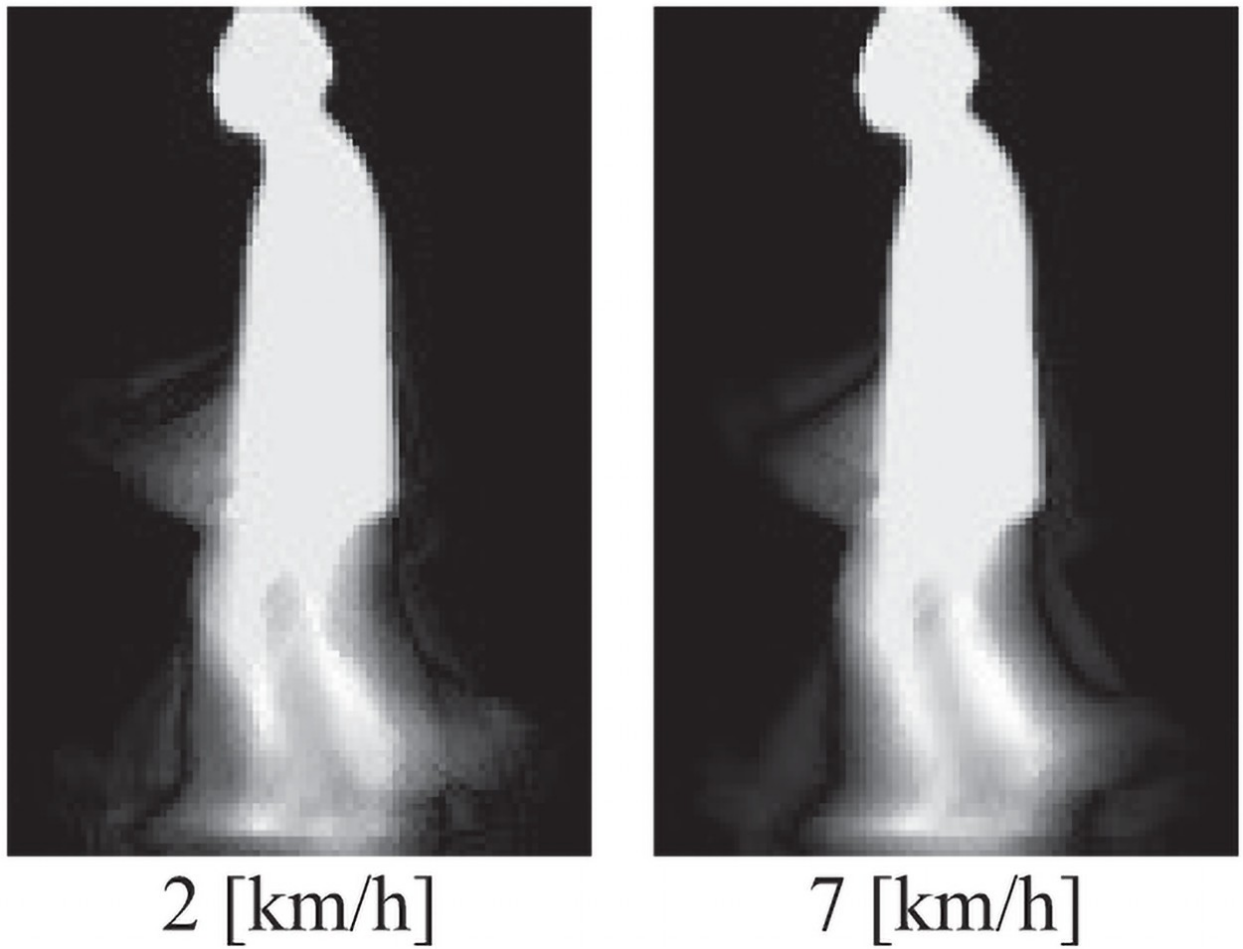
Visualization of two canonical vectors (2 [km/h] and 7 [km/h]) for the 1st canonical angle.

To show how the d-dimensional subspace method works, we visualize canonical vectors corresponding to each canonical angle defined in Eq 2. From the definition of Eq 2, the *r*th canonical angle can be regarded as an angle between the gallery canonical vector $v_{c,r}^{G}$ of class *c* and the input canonical vector $v_{c,r}^{P}$ of the subject. The input canonical vector $v_{c,r}^{P}$ is defined as $v_{c,r}^{P}=\sum_{d=1}^{d^{P}}\omega \left(r,d\right)\psi^{d}$, where *ω*(*r*, *d*) is an element of a matrix constructed from eigenvectors $\omega_{c}^{\left(r,d^{G},d^{P}\right)}$. The gallery canonical vector $v_{c,r}^{G}$ is also calculated in the same manner. Two canonical vectors of the 1st canonical angle represent the most similar vectors between the gallery and the subject images. Fig 4 shows visualizations of a gallery canonical vector and an input one for the 1st canonical angle, when gait images of 2 [km/h] and those of 7 [km/h] are used for gallery and input images, respectively. This suggests that MSM successfully extracts speed-invariant information, although GEIs show speed-variant information as shown in Fig 2(b) and 2(d).

## The enhanced mutual subspace method (eMSM)

In this section we explain our proposed approaches (2D-PCA MSM, 2D-PCA-Rotated MSM (2D-PCA-R MSM), and Boosted-2D-PCA-R MSM (B-2D-PCA-R MSM)).

### 2D PCA-based mutual subspace method (2D-PCA MSM)

First we explain 2D principal component analysis (2D PCA), followed by the proposed 2D-PCA MSM.

In 2D principal component analysis (PCA) [15] a covariance matrix is calculated directly from image matrices (i.e. not from 1D vectors, which are used in a regular PCA). Thus the size of the covariance matrix of 2D PCA is smaller than that of the PCA, and this results in higher accuracy of the covariance matrix. More details are given in the following.

Given $N_{c}^{G}$ images $I_{c,n}^{G}$ (*n* = 1, …, $N_{c}^{G}$), an *A* × *B* matrix (*A* and *B* are height and width of an image, respectively), in a gallery dataset, an image covariance matrix $S_{c}^{G}$ is calculated by

$$\begin{array}{c} S_{c}^{G}=\frac{1}{N_{c}^{G}}\sum_{n=1}^{N_{c}^{G}}{\left(I_{c,n}^{G}-\mu_{c}^{G}\right)}^{T}\left(I_{c,n}^{G}-\mu_{c}^{G}\right), \end{array}$$ (3)

where $\mu_{c}^{G}=\frac{1}{N_{c}^{G}}\sum_{n=1}^{N_{c}^{G}}I_{c,n}^{G}$. Eigenvectors of $S_{c}^{G}$ are obtained as $\eta_{c,1}^{G},\ldots ,\eta_{c,K}^{G}$ (the first *K* largest eigenvalues).

Instead of using 1D vectors of images as input vectors into MSM, the 2D-PCA MSM uses projected vectors by 2D PCA as input vectors into MSM. Each image $I_{c,n}^{G}$ is projected with eigenvectors $\eta_{c,k}^{G}$ (1 ≤*k* ≤ *K*) and a projected vector $\xi_{c,n,k}^{G}$ is obtained by $\xi_{c,n,k}^{G}=I_{c,n}^{G}\eta_{c,k}^{G}$, and a set of projected vectors $\xi_{c,n,1}^{G},\ldots ,\xi_{c,n,K}^{G}$ are obtained. The same process is applied to each input image $I_{n}^{P}$ and a projected vector $\xi_{c,n,k}^{P}$ is obtained by $\xi_{c,n,k}^{P}=I_{n}^{P}\eta_{c,k}^{G}$. From the projected vectors, we define a gallery projected matrix and an input projected matrix as $\Xi_{c,n}^{G}=\left[\xi_{c,n,1}^{G},\ldots ,\xi_{c,n,K}^{G}\right]$ and $\Xi_{c,n}^{P}=\left[\xi_{c,n,1}^{P},\ldots ,\xi_{c,n,K}^{P}\right]$, respectively.

**Fig 5:**
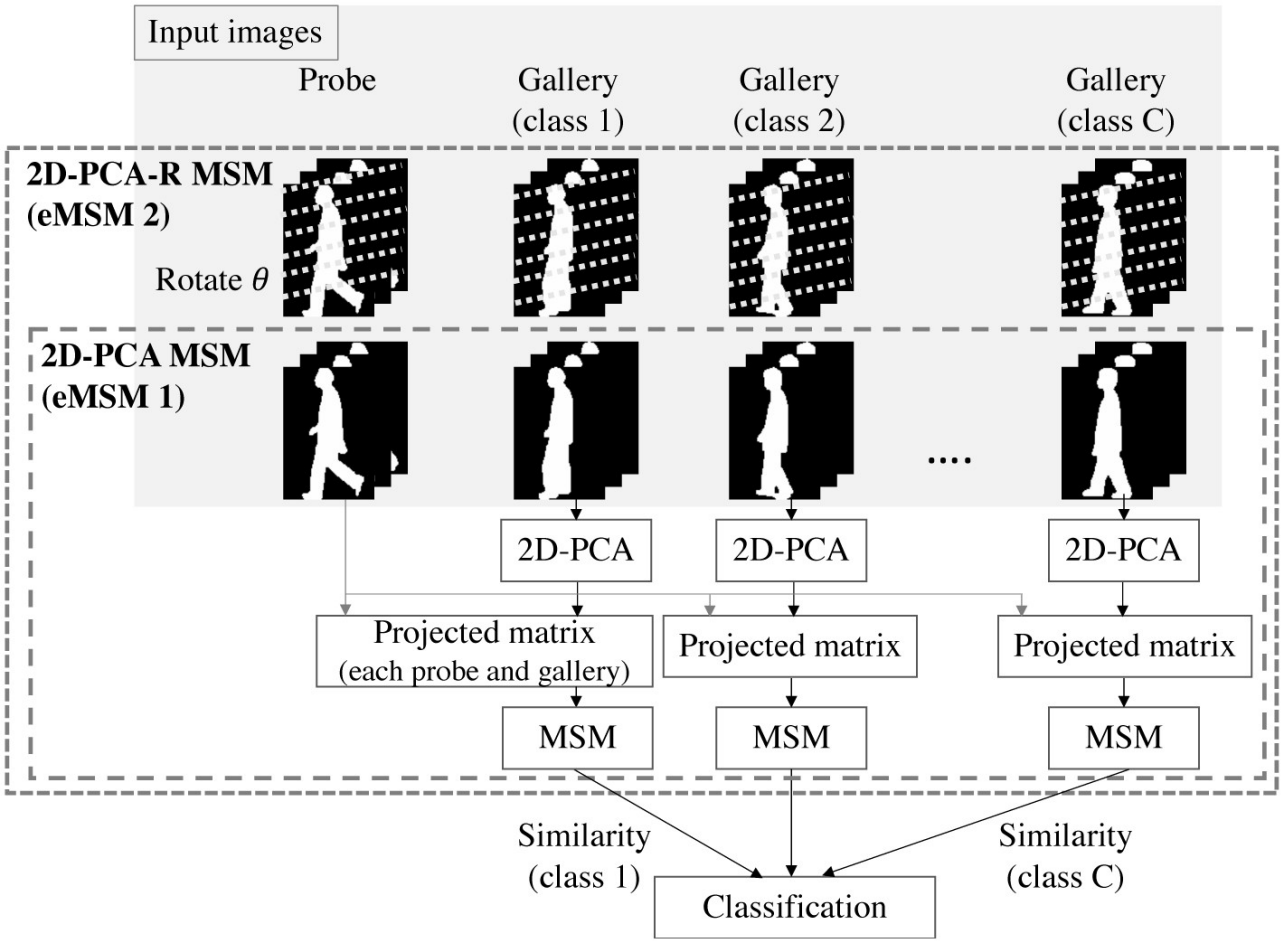
Overview of 2D-PCA MSM (smaller dotted rectangle) and 2D-PCA-R MSM (larger dotted rectangle).

The set of projected matrices $\Xi_{c,1}^{G},\ldots ,\Xi_{c,N_{c}^{G}}^{G}$ of the class *c* in the gallery dataset and the set of projected matrices $\Xi_{c,1}^{P},\ldots ,\Xi_{c,N^{P}}^{P}$ in the probe dataset are used as input for MSM. Here, each of the projected matrices is an *A* × *K* matrix. Similarities (i.e. eigenvalues) $s_{c}^{\left(r,d^{G},d^{P}\right)}$ between the probe dataset and each class *c* of the gallery datasets are calculated, followed by classification. In Fig 5, the smaller dotted rectangle shows the overview of the process of the proposed 2D-PCA MSM.

The size of the projected matrices is smaller than that of those original images, where the projected matrices represent gait images efficiently. Thus the use of projected matrices as input into MSM has the potential to produce higher performance compared with the use of the original images.

To show the effectiveness of 2D PCA compared with PCA, we performed preliminary experiments on the OU-ISIR Treadmill Dataset A. Our experiment did matching between gait images of 7 km/h (gallery) and those of 2 km/h (probe) as shown in Fig 2. We build a classifier *h* as

$$\begin{array}{c} h^{\left(r,d^{G},d^{P}\right)}=\underset{c}{\text{arg}\;\text{max}}\;s_{c}^{\left(r,d^{G},d^{P}\right)}. \end{array}$$ (4)

**Fig 6:**
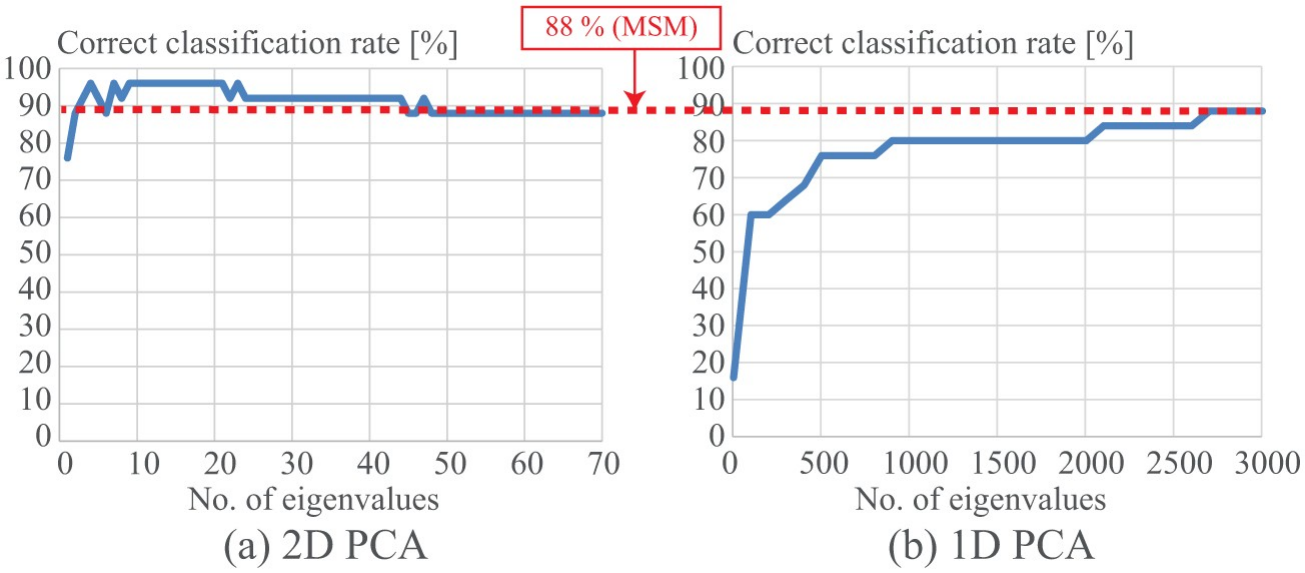
(a) Correct classification rate (CCR) with respect to the change of the number of eigenvalues of 2D PCA and (b) CCR of the PCA.

In the following examples, we set *r* = 1 (i.e., maximum eigenvalue), and *d*^*G*^ and *d*^*P*^ are defined by cross validation experiments. As a baseline result, we obtained a 88% correct classification rate by MSM. Fig 6(a) shows results of the matching by the proposed 2D PCA-based MSM with respect to changes of the number of eigenvalues *K* of 2D PCA, and we can see that the number of eigenvalues *K* between 9 and 21 shows improved performance over MSM. Fig 6(b) shows results from the PCA-based MSM with respect to the number of eigenvalues of PCA with the highest performance of 88% in case that the number of eigenvalues is over 2700. Here, in the PCA-based MSM, 1D-PCA is applied instead of the 2D-PCA used in Fig 5. These results show that 2D PCA-based MSM shows better performance than the PCA-based MSM.

**Fig 7:**
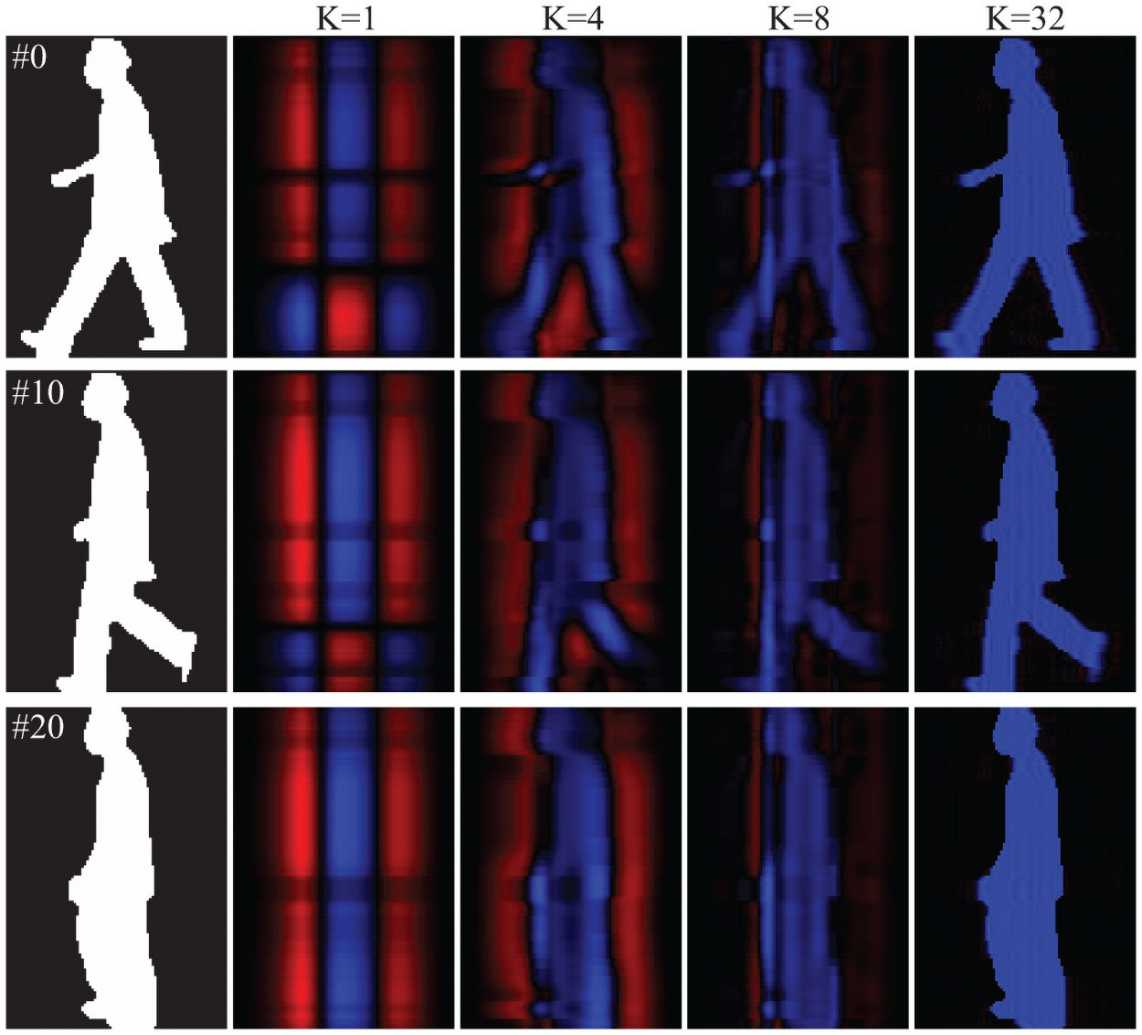
Examples of reconstructed images with *K* = 1, 4, 8, 32 by 2D PCA. Blue and red colors min positive and negative values.

**Fig 8:**
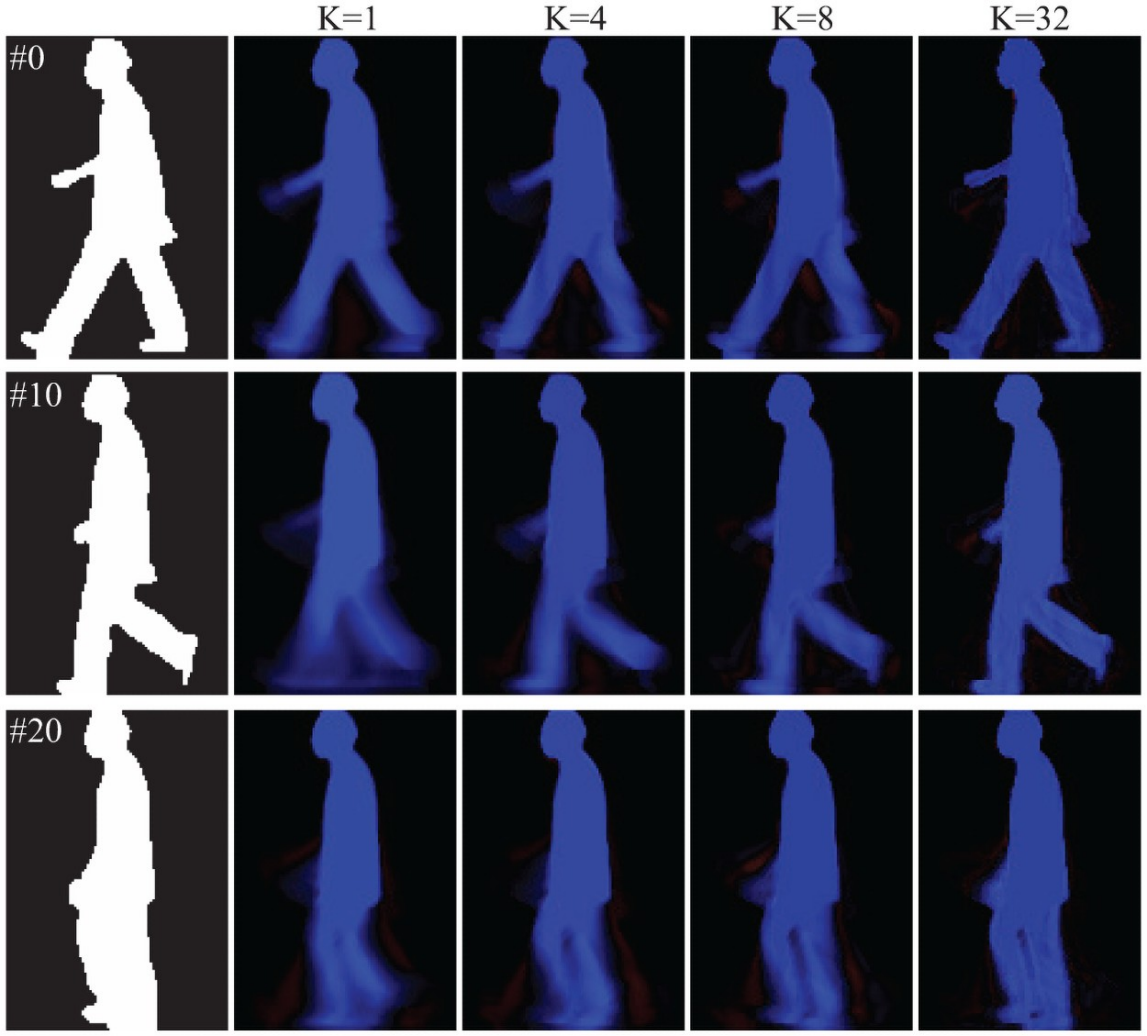
Examples of reconstructed images with *K* = 1, 4, 8, 32 by the PCA.

In 2D PCA, images $I_{c,n}^{G}$ can be reconstructed as ${\tilde{I}}_{c,n}^{G}=\sum_{k=1}^{K}\xi_{c,n,k}^{G}{\eta_{c,k}^{G}}^{T}$. Fig 7 shows reconstructed images with *K* = 1, 4, 8, 32 by 2D PCA. Using PCA, images can be also reconstructed in the same manner. Fig 8 shows reconstructed images with *K* = 1, 4, 8, 32 using PCA. The reconstructed images contain positive and negative values, which are shown in blue and red, respectively. In Fig 6(b) the performance by PCA-based MSM is not good especially in the case of *K* < 2700. One reason for such poor performance, as shown in Fig 8, is the reconstructed images with *K* = 1 contain blurred regions, especially in the leg and arm regions. This is a direct result of less accurate evaluation results of the covariance matrix computed by 1D PCA. On the other hand, in Fig 7 the reconstructed images with *K* = 1 by 2D PCA represent simplified information of the subject, such as the length of the legs and the location of hand/arm. Based on the accuracy shown in Fig 6(a), the performance with *K* = 1 by 2D PCA-based MSM is better than that by 1D PCA-based MSM, suggesting that the simplified information extracted using 2D PCA is more suitable for people recognition than the blurred information produced by 1D PCA.

### 2D-PCA MSM with Rotated Images (2D-PCA-R MSM)

2D PCA has been proven to be a special case of block-based PCA [25]. For example, blocks are horizontal lines of images. This suggests that the covariance matrix produced by 2D PCA from images is different from that of images rotated at a certain angle.

**Fig 9:**
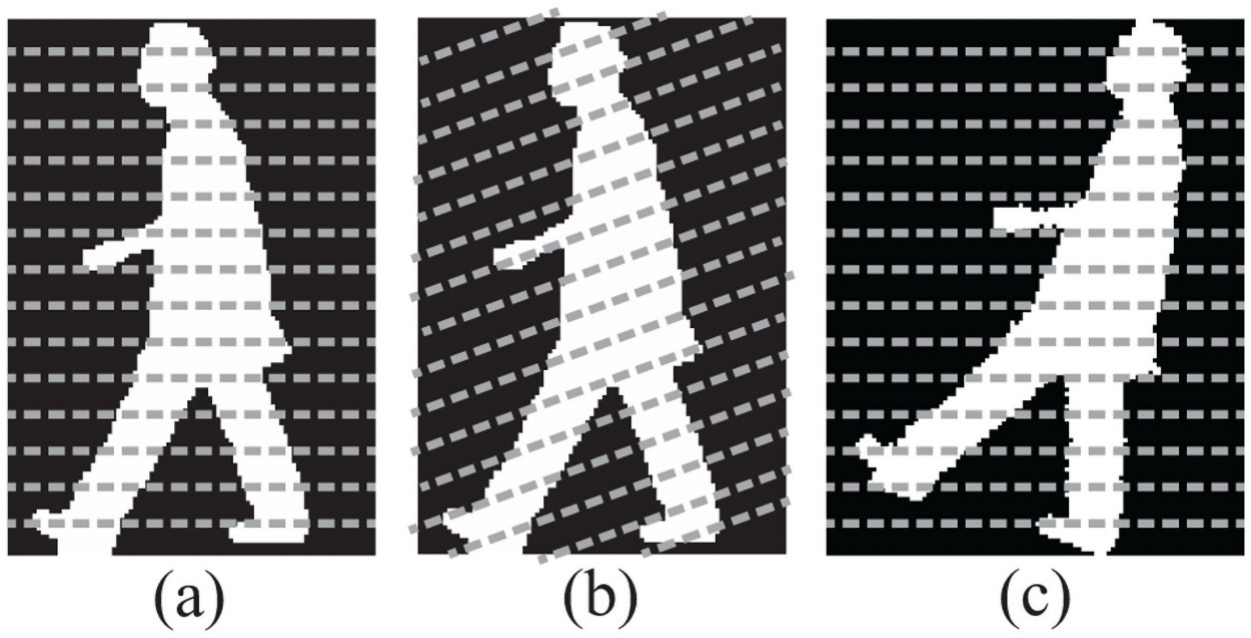
(a) An example original image, (b) image with scanning incident lines at 20 degree, and (c) 20 degree rotated image. (b) and (c) are equivalent from the point of view of resulting intersections of lines with body.

Fig 9(a) shows horizontal lines, which are used in 2D-PCA MSM. In the case that the lines are rotated at 20 degree as shown in Fig 9(b), the covariance matrix calculated from rotated parallel lines is different from that with horizontal lines. This results in extracting gait features which are different from those obtained using horizontal lines. Moreover, the horizontal lines rotated at a certain angle capture local biometric information, such as lengths of arms and leg segments, which are not captured by the horizontal lines. Therefore, similarities from lines rotated with various rotation angles contain richer gait/biometric features than those from the original images. Fusing similarities of lines rotated with various rotation angles is thus expected to improve the performance of gait recognition.

Because the implementation is simpler, in this paper we rotate an original image (Fig 9(a)) with respect to the optical axis of a camera instead of rotating the horizontal lines, and we obtain a rotated image as shown in Fig 9(c). This is equivalent to using rotated parallel lines on non-rotated images.

**Fig 10:**
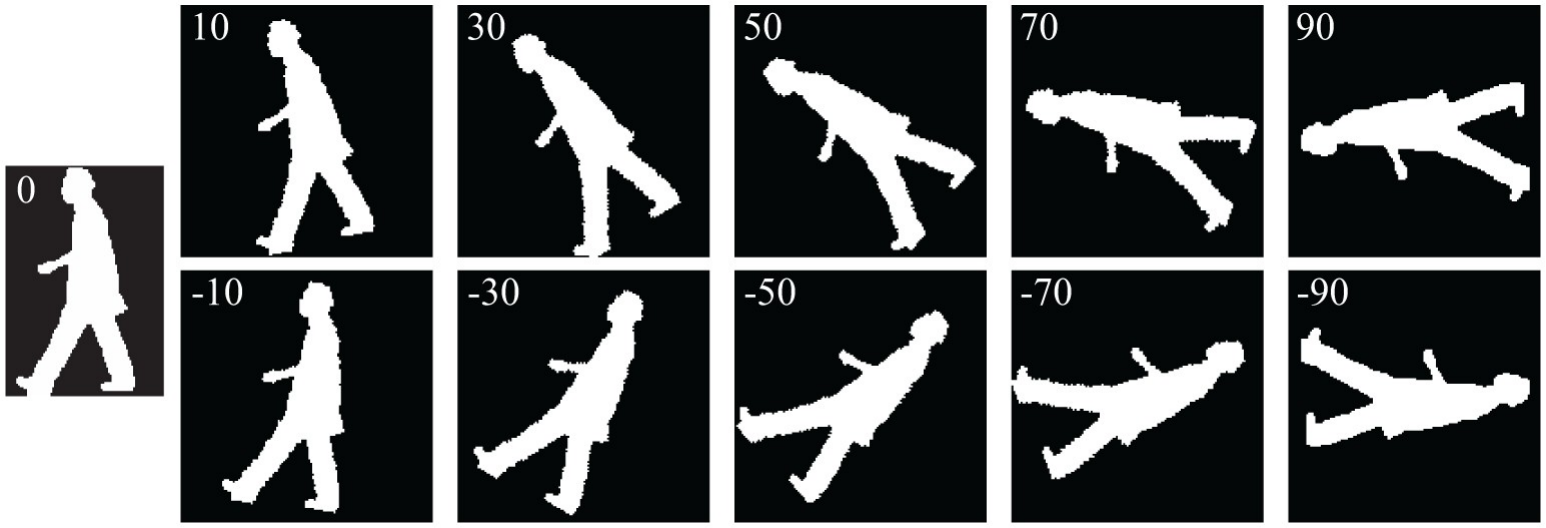
Examples of rotated images (0, 10, 30, 50, 70, 90, -10, -30, -50, -70, -90 degree).

In the 2D-PCA-R MSM, we rotated images *θ* [degree] (−Θ ≤ *θ* ≤ Θ, every Δ*θ*). Similarities $s_{c}^{\left(r,d^{G},d^{P},\theta \right)}$ between the probe dataset and each class *c* of the gallery datasets at every rotation angle *θ* are calculated. A classifier *h* is updated as $h^{\left(r,d^{G},d^{P},\theta \right)}=\underset{c}{\text{arg}\;\text{max}}\;s_{c}^{\left(r,d^{G},d^{P},\theta \right)}$. Fig 10 shows examples of rotated images (0, 10, 30, 50, 70, 90, -10, -30, -50, -70, and -90 degree). In Fig 5, the larger dotted rectangle shows the overview of the process of the proposed 2D-PCA-R MSM.

### Boosted-2D-PCA-R MSM (B-2D-PCA-R MSM)

There are various methods to fuse similarities, such as summation and voting. It is reported that the boosting method efficiently fuses the similarities of various canonical angles [26]. As discussed in section of MSM, if the dimensionalities *d*^*G*^ and *d*^*P*^ are changed, the recognition accuracy also changes. It may vary with changes of rotation angles. In general, these parameters are defined by cross validation experiments. In this paper we propose to fuse similarities from various dimensionalities, rotation angles, and canonical angles.

There are in total 4 parameters: canonical angle *r* (1 ≤ *r* ≤ *R* where *R* is the maximum number of canonical angles), dimensionalities *d*^*G*^ and *d*^*P*^ (1 ≤ *d*^*G*^ ≤ *D*^*G*^ and 1 ≤ *d*^*P*^ ≤ *D*^*P*^, where *D*^*G*^ and *D*^*P*^ are the dimensionalities of the gallery and input subspaces, respectively), and rotation angle *θ* (−Θ ≤ *θ* ≤ Θ, every Δ*θ*). Each combination of parameters produces different similarity, and the proposed method can efficiently choose combinations of parameters which improve the recognition accuracy.

To select parameters contributing to gait recognition, we use AdaBoost [27]. The main steps of the process are as follows:

**Step 1** The weight of each probe dataset is initialized as $D_{t=0}\left(i\right)=\frac{1}{N^{P}}$, where *i* is 1 ≤ *i* ≤ *N*^*P*^ (*N*^*P*^ is the number of probe datasets) and *t* is 1 ≤ *t* ≤ *T* (*T* is the maximum number of iterations in the boosting process).
**Step 2** At each classifier $h^{\left(r,d^{G},d^{P},\theta \right)}$, an error rate $\epsilon_{t}^{\left(r,d^{G},d^{P},\theta \right)}$ is calculated as
  $$\begin{array}{c} \epsilon_{t}^{\left(r,d^{G},d^{P},\theta \right)}=\sum_{i\in Failure(h^{\left(r,d^{G},d^{P},\theta \right)})}D_{t}\left(i\right). \end{array}$$ (5)
  This process is done for all classifiers (i.e. all combinations of parameters).
**Step 3** The classifier with the minimum $\epsilon_{t}^{\left(r,d^{G},d^{P},\theta \right)}$ among all classifiers is selected as $h_{t}^{p_{t}}$ at iteration *t*, and *p*_*t*_ is the selected combination of parameters of canonical angles, dimensionalities, and rotation angle at iteration *t*.
**Step 4** Confidence $\alpha_{t}\in R$ is calculated as
  $$\begin{array}{c} \alpha_{t}=\frac{1}{2}ln\frac{1-\epsilon_{t}^{p_{t}}}{\epsilon_{t}^{p_{t}}}. \end{array}$$ (6)
**Step 5** Weights *D*_*t*_(*i*) are updated as
  $$D_{t+1}\left(i\right)=\frac{D_{t}\left(i\right)exp\left(-\alpha_{t}h^{\prime }\left(i\right)\right)}{Z_{t}}$$ (7)
  $$Z_{t}=\sum_{i=0}^{N^{P}}D_{t}\left(i\right)exp\left(-\alpha_{t}h^{\prime }\left(i\right)\right),$$ (8)
  where
  $$\begin{array}{c} h^{\prime }\left(i\right)=\{\begin{array}{cc} 1 & h_{t}^{p_{t}}\left(i\right)\;\text{is}\;\text{correct}\\ -1 & \text{otherwise}. \end{array} \end{array}$$
**Step 6** Update *t* as *t* + 1 and repeat Steps 2∼5 if *t* < *T*.

Final classifier *H* is defined as

$$\begin{array}{c} H=\underset{c}{\text{arg}\;\text{max}}\sum_{t=1}^{T}score_{t,c}, \end{array}$$ (9)

where

$$\begin{array}{c} score_{t,c}=\{\begin{array}{cc} \alpha_{t} & h_{t}^{p_{t}}\;\text{is}\;\text{class}\;\text{c}\\ 0 & \text{otherwise}. \end{array} \end{array}$$

**Fig 11:**
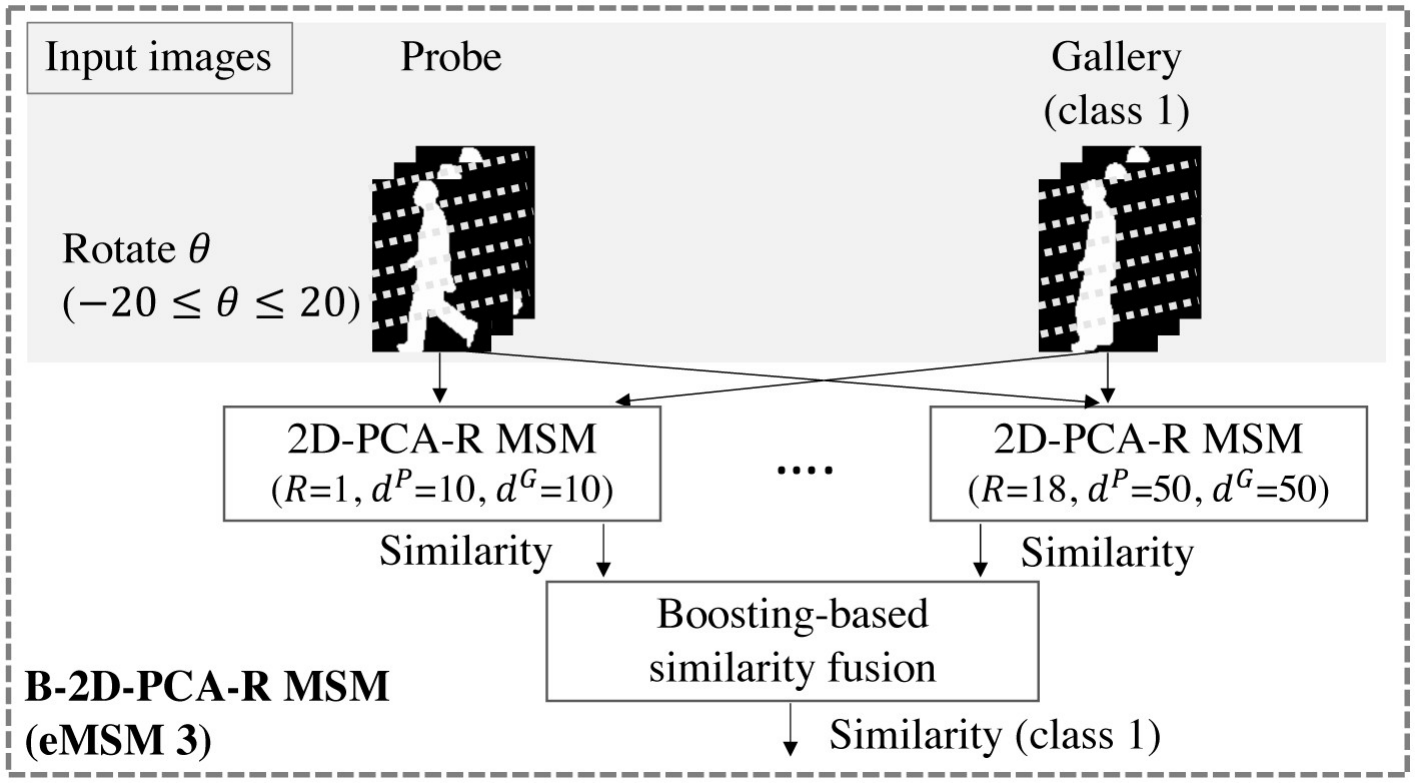
Overview of B-2D-PCA-R MSM. The output similarity is used in the classification process in Fig 5.

Fig 11 shows the overview process of the B-2D-PCA-R MSM. Similarities from various dimensionalities, rotation angles, and canonical angles are fused by the boosting method, followed by the classification process in Fig 5.

## Experiments

In this section we implement the 2D-PCA MSM, the 2D-PCA-R MSM, and the B-2D-PCA-R MSM, and we evaluate the performance of the proposed methods on gait databases. In our experiments we used the following 3 gait databases: (i) CASIA-C Dataset [14], (ii) OU-ISIR Treadmill Dataset A [13], and (iii) OU-ISIR Gait Speed Transition Dataset [8]. These data sets were chosen as they include speed variability. To check if the proposed eMSM correctly distinguishes datsets in which people are walking, and those in which do not (they perform other activities), we also tested against the KTH action dataset [28].

### Gait recognition with the CASIA-C Dataset

The CAISIA-C Dataset consists of 153 subjects with 3 different walking speeds and a carrying condition. Since this paper focuses on walking speed condition, we apply the proposed method to datasets of different walking speeds. The three walking conditions include normal walking (*fn*), slow walking (*fs*), and quick walking (*fq*). For each subject, there are 4 sequences of *fn*, 2 sequences of *fs*, and 2 sequences of *fq*. We used 3 normal walking (*fn*) sequences as the gallery set, and slow walking (*fs*) was used as the probe set. The image resolution is 320 × 240.

The following list shows 2 experiments. The first experiment is done to determine the proper ranges of 4 parameters *R*, *D*^*G*^, *D*^*P*^, and Θ. The second experiment was done to confirm the effectiveness of the B-2D-PCA-R MSM.

1) By the B-2D-PCA-R MSM, train (i) a parameter rotation angle *θ* while keeping the rest of parameters fixed, (ii) a parameter canonical angle *r* while fixing the rest, and (iii) parameters *d*^*G*^ and *d*^*P*^ while fixing the rest.
2) By the B-2D-PCA-R MSM, train all parameters *r*, *d*^*G*^, *d*^*P*^, and *θ*.

To fix parameters which are not trained (e.g. for the second experiment, the number of eigenvalues *K* of the 2D-PCA) and train the parameters, we used quick walking (*fq*) data in the case that slow walking (*fs*) data is used as the probe data, and vice-versa.

**Train (i) a parameter rotation angle *θ* while keeping the rest of parameters fixed, (ii) a parameter canonical angle *r* while fixing the rest, and (iii) parameters *d*^*G*^ and *d*^*P*^ while fixing the rest**.

**Fig 12:**
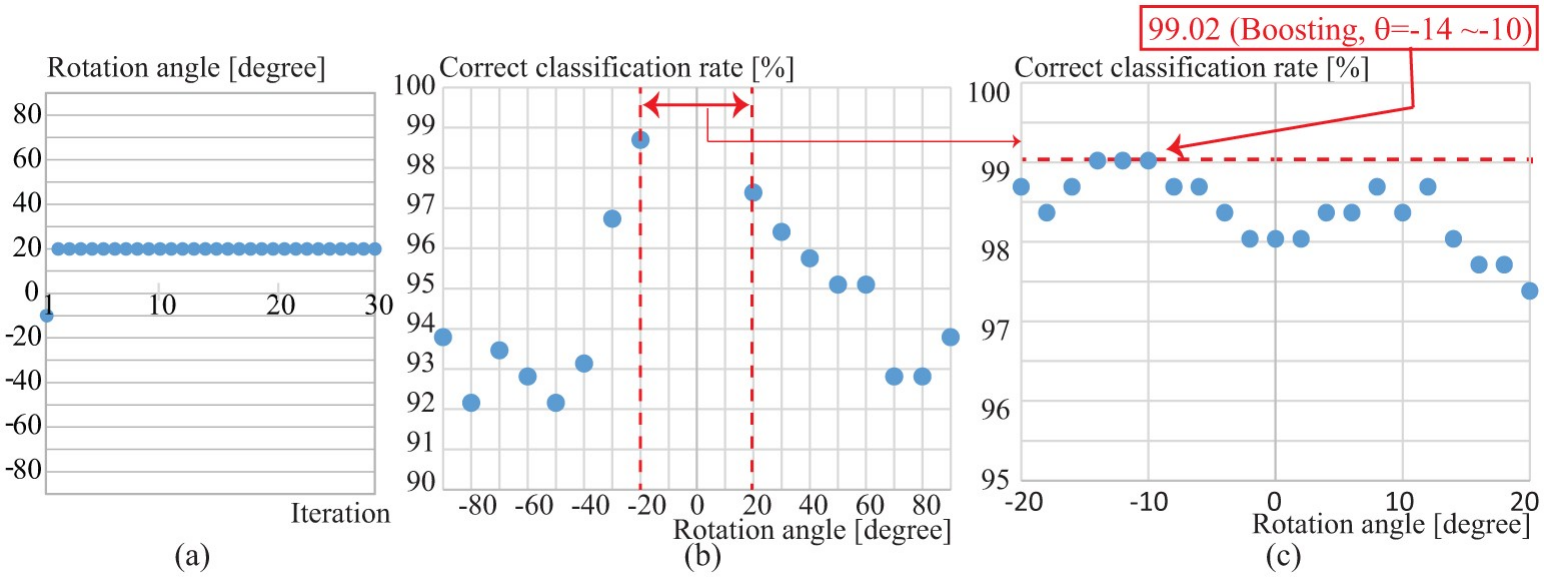
(a) Selected rotation angle at each iteration by the boosting method, (b) correct classification rate (CCR) at each rotation angle (-90 ≤ *θ* ≤ -20 and 20 ≤ *θ* ≤ 90), and (c) CCR at each rotation angle (-20 ≤ *θ* ≤ 20) (the dotted line shows the CCR by fusing all rotation angles by the boosting method).

First, we train a parameter rotation angle *θ* while keeping the rest of parameters fixed, to determine the range of parameter Θ. We used slow walking (*fs*) as probe data and we applied the boosting method to fuse similarities of all rotation angles from −Θ [degree] to Θ [degree]. Here, we set two different angle intervals. One is Δ*θ* = 10 for |*θ*| ∈ [20, 90] (-90 ≤ *θ* ≤ -20 and 20 ≤ *θ* ≤ 90), and the other one is Δ*θ* = 2 for -20 ≤ *θ* ≤ 20. We set the canonical angle *R* = 1 (i.e. the maximum eigenvalue). The correct classification rate by the B-2D-PCA-R MSM is 99.02%, and the trained parameters at each iteration during the boosting process is shown in Fig 12(a). At the first iteration -10 degrees is selected and after that 20 degrees continuously selected. None of rotation angles from -90 to -30 and from 30 to 90 is selected. Therefore in the following experiments we set Θ = 20 degrees.

We also evaluated the classifier *h*_(*θ*)_ at each *θ* (i.e. we used 2D-PCA-R MSM). Fig 12(b) and 12(c) show correct classification rates of |*θ*| ∈ [20, 90] (-90 ≤*θ*≤ -20 and 20 ≤*θ*≤ 90, *Δθ* = 10), and -20 ≤*θ*≤ 20 (Δ*θ* = 2), respectively. These results show that recognition accuracy is relatively high between -20 and 20 degrees. The highest recognition accuracy is 99.02%, which is the same performance as the B-2D-PCA-R MSM fusing multiple rotation angles (the red dotted line in Fig 12(c)). This suggests that in the case of the canonical angle *r* = 1, 2D-PCA-R MSM at each rotation angle does not compensate each other and works as a strong classifier.

The result of *θ* = 0 degree is the result produced by the 2D-PCA MSM. Table 1(a)–1(c) shows the correct classification rates of MSM, 2D-PCA MSM, and 2D-PCA-R MSM. From these results, the effectiveness of the proposed 2D-PCA-R MSM is confirmed.

**Table 1:** Comparison of correct classification rate (CCR) [%] of each of MSM [12], the proposed 2D-PCA MSM, the proposed 2D-PCA-R MSM (*θ* = −10), and the proposed B-2D-PCA-R MSM on CASIA-C [14] (slow walk *fs*).

Methodology	CCR
(a) MSM	97.06
(b) 2D-PCA MSM	98.04
(c) 2D-PCA-R MSM (*θ* = -10)	99.02
(d) B-2D-PCA-R MSM	**100.0**

**Fig 13:**
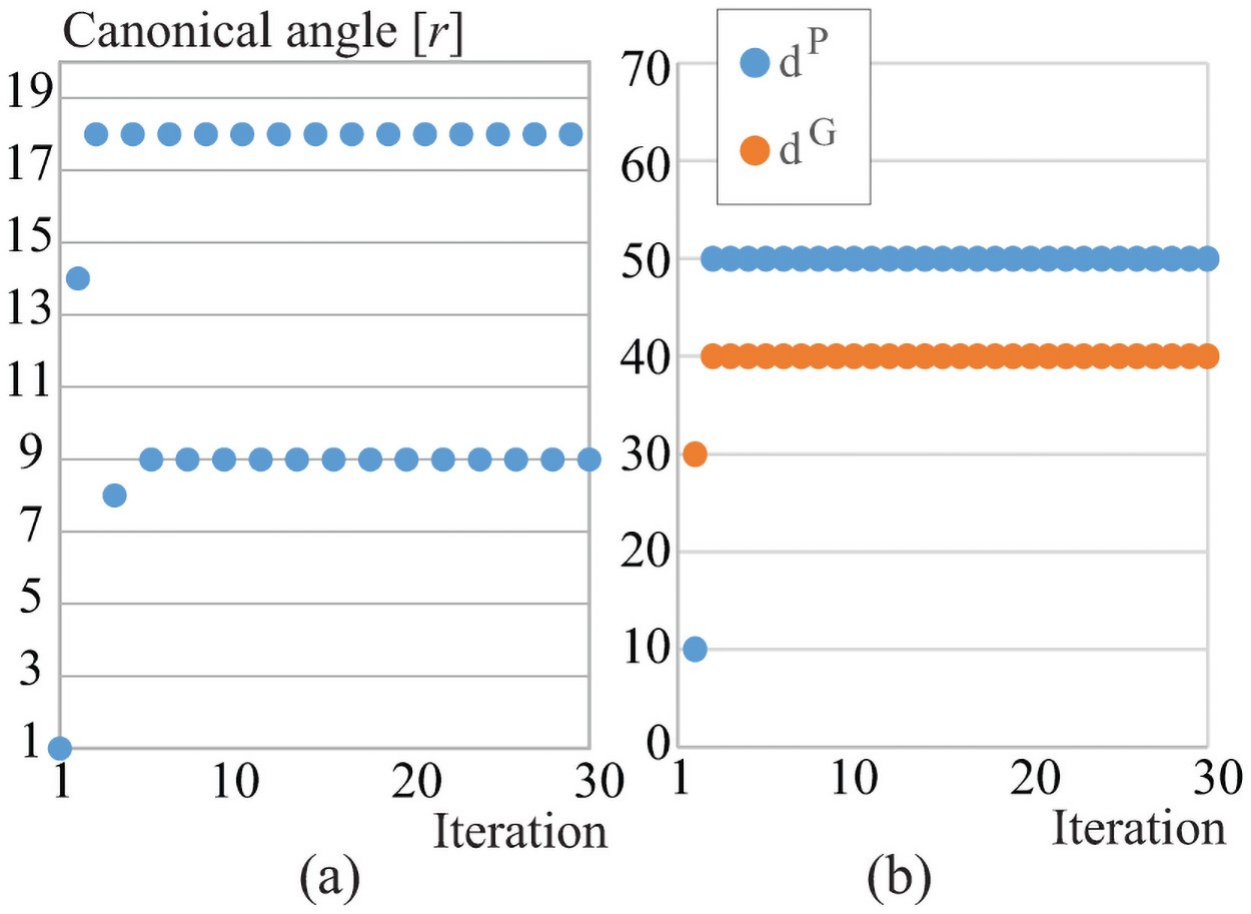
(a) Selected canonical angle *r* at each iteration by the boosting method, and (b) selected *d*^*G*^ and *d*^*P*^ at each iteration by the boosting method.

Next, we train a parameter canonical angle *r* while keeping the rest of parameters fixed to determine the range of parameter *R*. We set *R* = 20 and rotation angle Θ = 0. The correct classification rate by the boosting method is 99.35%, and Fig 13(a) shows the trained canonical angles at each iteration in the boosting process. Based on this result, in the following experiments we set the parameter canonical angle *R* as 18.

Lastly, to fix the parameters *D*^*G*^ and *D*^*P*^, we set *d*^*G*^ and *d*^*P*^ from 10 to 70 (every 10), respectively. We set the rotation angle and the canonical angle as Θ = 0 and *R* = 1, respectively. The correct classification rate by the boosting method is 98.7%, and Fig 13(b) shows the trained canonical angles. Based on this result in the following experiments we set the parameters *D*^*G*^ and *D*^*P*^ as 50.

#### Select all parameters *r*, *d*^*G*^, *d*^*P*^, and *θ*

**Fig 14:**
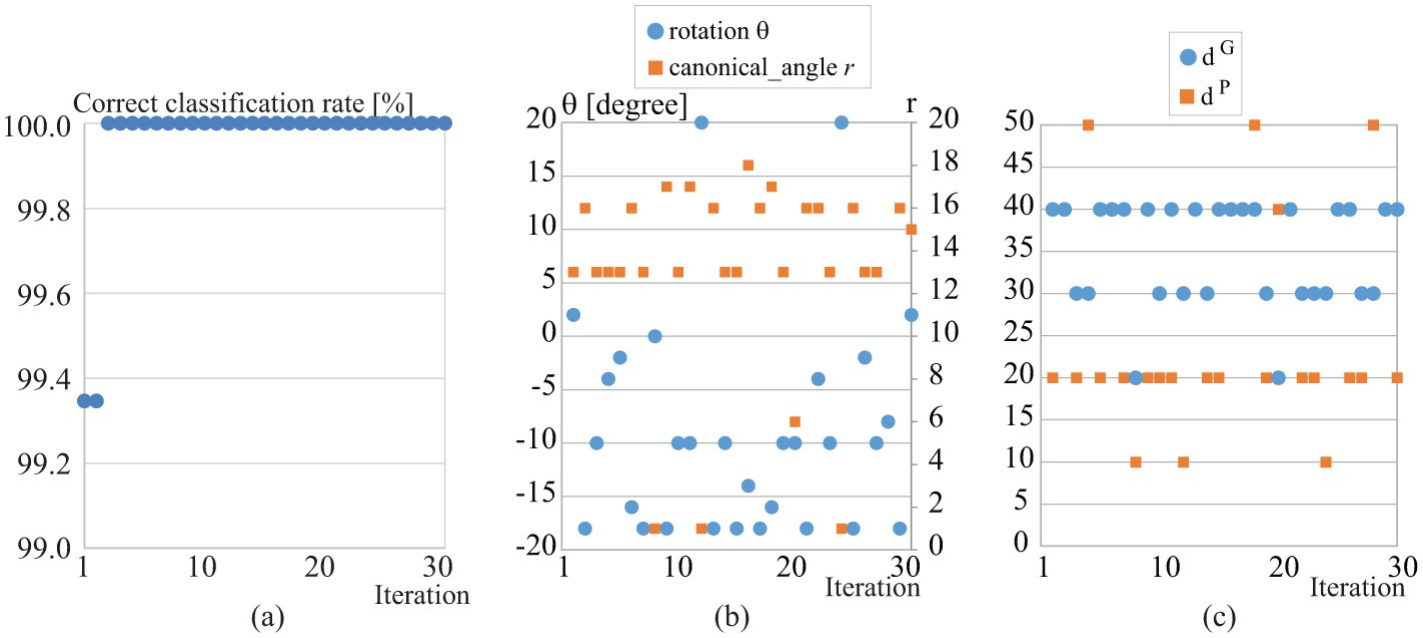
(a) Correct classification rate (CCR) at each iteration by applying the boosting method to similarities of all parameters (rotation angles, canonical angles, and dimensionalities), (b) selected canonical angle *r* and rotation angle *θ*, and (c) selected *d*^*G*^ and *d*^*P*^.

In the last experiment we fused similarities of all parameters *r*, *d*^*G*^, *d*^*P*^, and *θ*. We set canonical angle *r* from 1 to 18, *d*^*G*^ and *d*^*P*^ from 10 to 50 at every 10, and rotation angle *θ* from -20 degree to 20 degree at 2 degree increments. The correct classification rate is 100.0% after 3 iterations as shown in Fig 14(a), and the use of full 4 parameters in the B-2D-PCA-R MSM produces the highest accuracy (100%) as shown in Table 1(d). This result shows that B-2D-PCA-R MSM is robust to speed changes. Fig 14(b) and 14(c) show the selected 4 parameters. Here, robustness to variation of walking speed means that one maintains high accuracy of classification for a diversity of speeds of the walking individuals in different datasets; many other methods are sensitive to such speed variability, which reduces the accuracy.

### Comparison with state-of-the-art methods

#### Gait recognition with the CASIA-C Dataset

As performed in the previous section, we used normal walking (*fn*) sequences as the gallery set, and both slow walking (*fs*) and quick walking (*fq*) sequences in the probe set. Table 2 shows the correct classification rates by the B-2D-PCA-R MSM, the spatiotemporal motion-based method [19], the RSM-based gait recognition [17], and the MSM-based method [12], and single-support GEI (SSGEI) [18]. These results show the effectiveness of the proposed method.

**Table 2:** Comparison of the Correct Classification Rate (CCR) [%] of each of the B-2D-PCA-R MSM, the RSM-based gait recognition [17], the motion-based method [19], the MSM-based method with divided areas [12], and single-support GEI (SSGEI) [18] on CASIA-C [14] (slow walk *fs* and quick walk *fq*).

Methodology	*fs*	*fq*
B-2D-PCA-R MSM	**100.0**	**100.0**
RSM-based method [17]	99.7±0.24	99.6±0.14
Motion-based method [19]	99.4	**100.0**
MSM-based method [12] with divided areas	99.7	99.7
SSGEI [18]	99.5±0.61	99.5±0.53

#### Gait recognition with the OU-ISIR Treadmill Dataset A

The OU-ISIR Treadmill Dataset A consists of gait images of 34 people, each with a gallery dataset and a probe dataset. From the dataset specification, 25 subjects are assigned for evaluation while the rest are used for parameter training, where the image resolution is 128 × 88. This dataset consists of gait images with speed variations (from 2 km/h to 10 km/h, every 1 km/h) for each gallery and probe dataset. Subjects walked for speeds between 2 km/h to 7 km/h and run for speeds between 8 km/h to 10 km/h. In our experiments, we used images with speeds between 2 km/h to 7 km/h, and we performed experimental evaluations with all combinations of gallery and probe speeds, i.e. 36 combinations (“6 different speed for each gallery dataset” × “6 different speeds for each probe dataset”).

Table 3 lists the average classification rates of our B-2D-PCA-R MSM (99.89%), the MSM-based method with divided areas [12] (99.78%), and the SSGEI-based method [18] (99.33%). In Table 4, bold numbers and numbers in round brackets show results of cross-speed walking people identification with our B-2D-PCA-R MSM and the MSM-based method with divided areas [12], respectively. Our method shows slightly better performance than the MSM-based method [12]. Furthermore, the equal error rate (EER) by the proposed method is greatly improved compared with the MSM-based method with divided areas [12]. In the case that the absolute speed difference between the gallery and probe data is less than 2 [km/h], average EER is 0 for both the B-2D-PCA-R MSM and the MSM-based method with divided areas [12], as shown in Table 5. In the case that the absolute speed difference is greater than or equal to 2 [km/h], average EER gradually increases for the MSM-based method with divided areas [12], where the maximum averaged EER is 3.0%. On the contrary, the maximum average EER for the B-2D-PCA-R MSM is 0.05%.

**Table 3:** Comparison of average correct classification rate (CCR) [%] of each the proposed B-2D-PCA-R MSM, MSM-based method with divided areas [12], and SSGEI-based method [18].

	B-2D-PCA-R MSM	MSM-based method [12] with divided areas	SSGEI [18]
CCR [%]	**99.89**	**99.78**	99.33

**Table 4:** CCR [%] of the B-2D-PCA-R MSM and the MSM-based method with divided areas [12] (numbers in round brackets) in the cross-speed walking gait recognition.

Probe	2 km/h	3 km/h	4 km/h	5 km/h	6 km/h	7 km/h
Gallery						
2 km/h	100 (100)	100 (100)	100 (100)	100 (100)	100 (100)	**96** (92)
3 km/h	100 (100)	100 (100)	100 (100)	100 (100)	100 (100)	100 (100)
4 km/h	100 (100)	100 (100)	100 (100)	100 (100)	100 (100)	100 (100)
5 km/h	100 (100)	100 (100)	100 (100)	100 (100)	100 (100)	100 (100)
6 km/h	100 (100)	100 (100)	100 (100)	100 (100)	100 (100)	100 (100)
7 km/h	100 (100)	100 (100)	100 (100)	100 (100)	100 (100)	100 (100)

**Table 5:** Averaged equal error rate (EER) [%] at each absolute speed difference between gallery and probe data for the proposed B-2D-PCA-R MSM and the MSM-based method with divided areas [12].

Methodology	Absolute speed difference [km/h]					
	0	1	2	3	4	5
B-2D-PCA-R MSM	0.00	0.00	**0.00**	**0.00**	**0.00**	**0.05**
MSM-based method with divided area [12]	0.00	0.00	0.04	0.20	0.93	3.00

**Fig 15:**
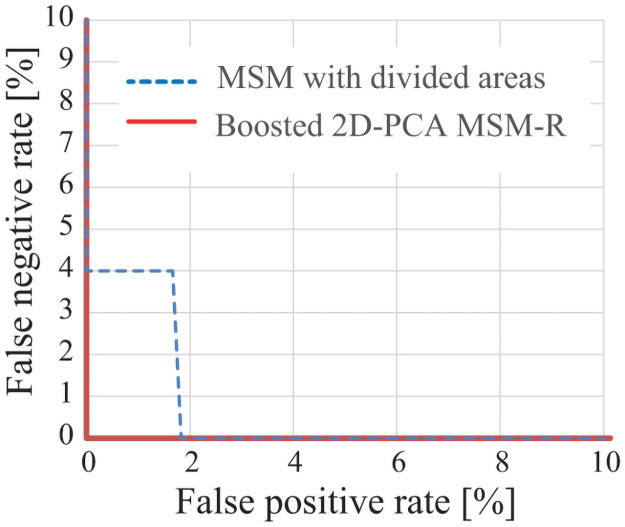
ROC curves of the MSM-based method with divided areas [12] and the B-2D-PCA-R MSM. Gallery speed and probe speed are 3 km/h and 7 km/h, respectively.

Fig 15 shows the ROC (Receiver Operating Characteristic) curves between 3 km/h (gallery) and 7 km/h (probe) by the MSM-based method with divided areas [12] and the B-2D-PCA-R MSM. Though this speed combination of gallery and probe data results in the same correct classification rates (100%) between the B-2D-PCA-R MSM and the MSM-based method with divided areas [12], the ROC curves show that the B-2D-PCA-R MSM is superior to the MSM-based method with divided areas.

#### Gait recognition with the OU-ISIR Gait Speed Transition Dataset

The OU-ISIR Gait Speed Transition Dataset consists of two different datasets, dataset 1 and dataset 2. In dataset 1 the probe set consists of speed transited gait sequences recorded from 26 subjects. The gallery dataset consists of gait sequences of 179 subjects, which include the 26 probe subjects, where the subjects walked at a constant speed (4 km/h) for a few seconds. In dataset 2 the probe set consists of 25 subjects where each subject walked twice on the treadmill under the following conditions: (i) accelerations from 1 km/h to 5 km/h and (ii) decelerations from 5 km/h to 1 km/h. For the gallery dataset, there are 154 subjects, which include the 25 probe subjects, where each subject walked at a constant speed (4 km/h) for six seconds. In addition to the two datasets we explained above, the OU-ISIR gait speed transition dataset has an auxiliary training set, which includes 24 subjects under various walking speeds (2, 3, 4, and 5 km/h). The parameters in our method were learned using the auxiliary training set which contains images of resolution 32 × 22.

We perform evaluation as follows. A set of images for the duration of one gait cycle are used for the evaluation of both gallery and probe datasets. For dataset 2, we divided each gait sequence into multiple gait sequences so that each of them includes gait images for the duration of one gait cycle. This is the same evaluation setting of [8].

**Fig 16:**
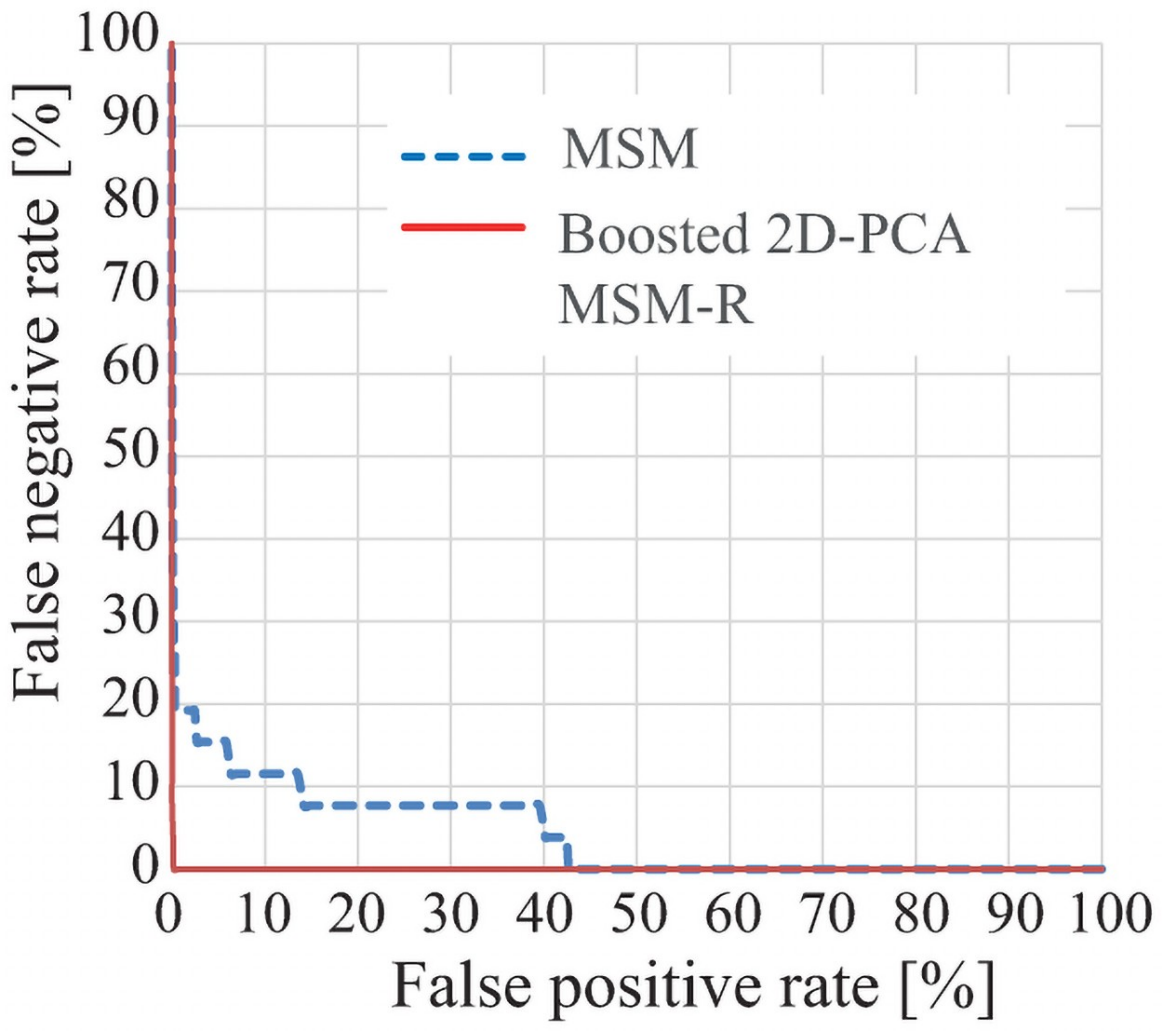
ROC curve by the B-2D-PCA-R MSM and the MSM-based method [11] for dataset 1.

Table 6 shows EER (equal error rate) and CCR (correct classification rate) of the MSM-based method [11] and the B-2D-PCA-R MSM for dataset 1. Here, we did not apply the MSM-based method with divided areas [12] due to the low image resolution, which causes performance degradation. Fig 16 shows ROC curve. Here, since Mansur et al. reported ROC curves graphically [8], we cannot show their results on this paper. For more details, please refer to [8]. In [11], we reported that the MSM-based method [11] outperformed the Mansur’s method [8]. The results in Table 6 and Fig 16 shows the performance of the B-2D-PCA-R MSM is better than that of the MSM-based method.

**Table 6:** Equal error rate (EER) and correct classification ratio (CCR) of MSM-based method [11] and the B-2D-PCA-R MSM, for dataset 1.

	MSM [11]	B-2D-PCA-R MSM
EER [%]	11.5	**0.2**
CCR [%]	84.6	**88.5**

Table 7 shows EER and CCR of Mansur’s method [8], the MSM-based method [11], and the B-2D-PCA-R MSM for dataset 2 accelerations and decelerations. From these results, it is clear that the B-2D-PCA-R MSM method outperformed existing methods.

**Table 7:** EER and CCR of Mansur’s method [8], MSM-based method [11] and the B-2D-PCA-R MSM, for dataset 2.

	Acceleration			Deceleration		
	Mansur [8]	MSM [11]	B-2D-PCA-R MSM	Mansur [8]	MSM [11]	B-2D-PCA-R MSM
EER [%]	8.0	7.0	**0.0**	8.0	6.0	**0.0**
CCR [%]	72.0	92.0	**100.0**	84.0	96.0	**100.0**

### Experiments with KTH action dataset

In this section we used the KTH action dataset [28] to evaluate if the proposed eMSM is able to correctly detect the cases when subjects are not walking, but doing something else (we refer to this as “not-walking” data). The KTH action dataset includes data from six activities (walking, jogging, running, boxing, hand waving and hand clapping) with 25 subjects, separated into train, validation, and test data (7, 7, and 8 subjects, respectively). Each subject has 4 data sets.

**Fig 17:**
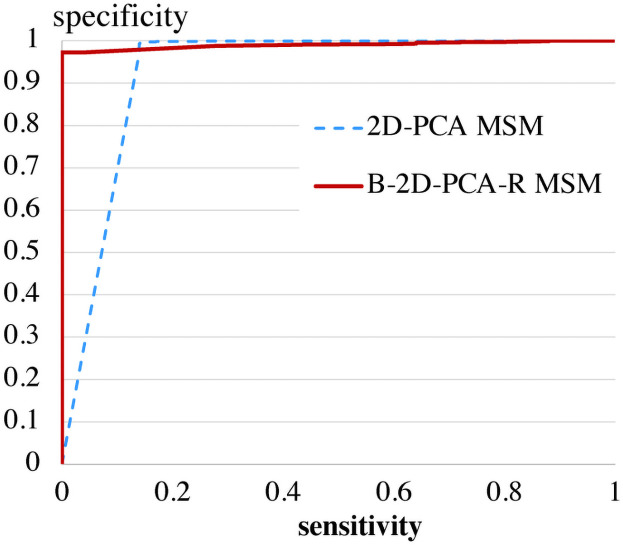
Specificities and sensitivities (defined as *TN* / (*TN* + *FP*) and *FN* / (*FN* + *TP*), respectively) by changing thresholds of 2D-PCA MSM and B-2D-PCA-R MSM.

To evaluate eMSM with the KTH dataset, we calculated specificities and sensitivities (defined as *TN* / (*TN* + *FP*) and *FN* / (*FN* + *TP*), respectively) by changing thresholds of the eMSM. As for the training data, we used OU-ISIR Treadmill Dataset A (gallery 2 [km/h]), and we used combination of OU-ISIR Treadmill Dataset A (probe 7 [km/h]) and KTH dataset as test dataset. The reason why we included the OU-ISIR Treadmill Dataset A in test data is to avoid denominators being 0 during the calculation of specificities and sensitivities. We used all KTH test dataset (in total 192 data sets (= 6×8×4)) in addition to OU-ISIR Treadmill Dataset A probe data (25 data sets). Fig 17 shows results by 2D-PCA MSM and B-2D-PCA-R MSM. The result of B-2D-PCA-R MSM shows very close to the left top area, which means that the B-2D-PCA-R MSM is robust in “not-walking” data sets.

## Conclusion

We proposed an enhanced mutual subspace method (eMSM) for gait recognition that is robust to variations in walking speed. The proposed method has three operational steps using these specific methods consecutively: First, a 2D PCA is utilized to calculate the covariance matrix with high accuracy, which enables us to represent subspaces of the original images efficiently. Second, rotated images are introduced to extract rich gait information. Third, boosting is applied to adaptively fuse multiple gait features. We carried out experiments using three public gait databases (CASIA-C Dataset, OU-ISIR Treadmill Dataset A, and OU-ISIR Gait Speed Transition Dataset), and we demonstrated that the proposed method outperformed state-of-the-art methods.

There are other operations to improve the performance, such as Random-Forest-based feature fusion and flipping images to extract rich gait features. These are left as future work.

The proposed method showed advantages in gait recognition with speed variation, which is one of appearance changes. It has the potential to be used for other types of appearance changes, such as cloth variation. Thus in the future we will try to develop a method which recognizes people with different clothes and speed.

## References

[1] Bouchrika I. and Goffredo M. and Carter J. and Nixon M. On Using Gait in Forensic Biometrics. J. of Forensic Sciences, 2011; 56(4):882–889. doi: 10.1111/j.1556-4029.2011.01793.x 21554307

[2] Han J. and Bhanu B. Individual recognition using gait energy image. Trans. Pattern Anal. Mach. Intell., 2006;28(2):316–322. doi: 10.1109/TPAMI.2006.38 16468626

[3] Y. Iwashita and R. Kurazume. Person identification from human walking sequences using affine moment invariants. IEEE Int. Conf. Robotics and Automation, 2009;436–441.

[4] Zhang E. and Zhao Y. and Xiong W. Active energy image plus 2dlpp for gait recognition. Signal Process, 2010;90(7):2295–2302. doi: 10.1016/j.sigpro.2010.01.024

[5] Lam T. and Cheung K. and Liu J. Gait flow image: A silhouette-based gait representation for human identification. Pattern Recognition, 2011;44(4):973–987. doi: 10.1016/j.patcog.2010.10.011

[6] M. Shinzaki and Y. Iwashita and R. Kurazume and K. Ogawara. Gait-Based Person Identification Method Using Shadow Biometrics for Robustness to Changes in the Walking Direction. IEEE Winter Conf. on Applications of Computer Vision, 2015;670–677.

[7] Tanawongsuwan R. and Bobick A. Modelling the effects of walking speed on appearance-based gait recognition. Computer Vision and Pattern Recognition, 2004;2:783–790.

[8] Mansur A. and Makihara Y. and Aqmar R. and Yagi Y. Gait Recognition under Speed Transition. Computer Vision and Pattern Recognition, 2014;2521–2528.

[9] O. Yamaguchi and K. Fukui and K. Maeda. Face recognition using temporal image sequence. Int. Conf. on Automatic Face and Gesture Recognition, 1998;318-323.

[10] Maeda K. From the Subspace Methods to the Mutual Subspace Method. Computer Vision, 2010;135–156. doi: 10.1007/978-3-642-12848-6_5

[11] Y. Iwashita and H. Sakano and R. Kurazume. Gait Recognition Robust to Speed Transition using Mutual Subspace Method. Int. Conf. on Image Analysis and Processing, 2015;141–149.

[12] Y. Iwashita and M. Kakeshita and H. Sakano and R. Kurazume. Making gait recognition robust to speed changes using mutual subspace method. Int. Conf. on Robotics and Automation, 2017;2273–2278.

[13] Makihara Y. and Mannami H. and Tsuji A. and Hossain M.A. and Sugiura K. and Mori A. and et al. The OU-ISIR Gait Database Comprising the Treadmill Dataset. IPSJ Trans. on Computer Vision and Applications, 2012;4:53–62. doi: 10.2197/ipsjtcva.4.53

[14] CASIA Gait Database, http://www.cbsr.ia.ac.cn/english/GaitDatabases.asp.

[15] Yang J. and Zhang D. and Frangi A.F. and Yang J. Two-Dimensional PCA: A New Approach to Appearance-Based Face Representation and Recognition. IEEE Trans. Pattern Anal Mach Intell, 2004;26(1):131–137. doi: 10.1109/TPAMI.2004.1261097 15382693

[16] Kusakunniran W. and Wu W. and Zhang J. and Li H. Gait recognition across various walking speeds using higher order shape configuration based on a differential composition model. IEEE Trans Syst Man and Cybern Part B: Cybern, 2012;42(6):1654–1668. doi: 10.1109/TSMCB.2012.2197823 22665509

[17] Y. Guan and C. Li. A Robust Speed-Invariant Gait Recognition System for Walker and Runner Identification. IAPR Int. Conf. on Biometrics, 2013:1–8.

[18] Xu C. and Makihara Y. and Li X. and Yagi Y. and Lu J. Speed-Invariant Gait Recognition Using Single-Support Gait Energy Image. Multimedia Tools and Applications, 2019:1–28.

[19] M.H. Khan and M.S. Farid and M. Grzegorzek. Person identification using spatiotemporal motion characteristics. IEEE Int. Conf. on Image Processing (ICIP), 2017;166–170.

[20] Z. Zhang and L. Tran and X. Yin and Y. Atoum and X. Liu and J. Wan et al. Gait Recognition via Disentangled Representation Learning. IEEE Conf. on Computer Vision and Pattern Recognition, 2019;4710–4719.

[21] Wu Z. and Huang Y. and Wang L. and Tan T. A comprehensive study on cross-view gait based human identification with deep cnns. IEEE Trans. on Pattern Analysis and Machine Intelligence, 2016;1(1):1–10. 10.1109/TPAMI.2016.2545669 27019478

[22] Takemura N. and Makihara Y. and Muramatsu D. and Echigo T. and Yagi Y. On Input/Output Architectures for Convolutional Neural Network-Based Cross-View Gait Recognition. IEEE Trans. on Circuits and Systems for Video Technology (TCSVT), 2017;28(1):1–13.

[23] F. Chatelin. Veleurs propres de matrices. Masson (In French), 1988.

[24] Watanabe S. and Lambert P. and Kulikowski C. and Buxton J. Evaluation and selection of variables in pattern recognition. Tou J. T. (Ed.), Computer and Information Sciences II, Academic Press”, 1967;91–122.

[25] Wang L. and Wang X. and Zhang X. and Feng J. The equivalence of two-dimensional PCA to line-based PCA. Pattern Recognition Letters, 2005;26(1):57–60. doi: 10.1016/j.patrec.2004.08.016

[26] Kim T.K. and Arandjelovic O. and Cipolla R. Boosted manifold principal angles for image set-based recognition. Pattern Recognition, 2007;40(9):2475–2484. doi: 10.1016/j.patcog.2006.12.030

[27] Freund Y. and Schapire R.E. A Decision-Theoretic Generalization of on-Line Learning and an Application to Boosting. J. Computer and System Sciences, 1997;55(1):119–139. doi: 10.1006/jcss.1997.1504

[28] C. Schuldt and I. Laptev and B. Caputo. Recognizing Human Actions: A Local SVM Approach. ICPR, 2004.

